# Healthcare Monitoring Using Low-Cost Sensors to Supplement and Replace Human Sensation: Does It Have Potential to Increase Independent Living and Prevent Disease?

**DOI:** 10.3390/s23042139

**Published:** 2023-02-14

**Authors:** Zhuofu Liu, Vincenzo Cascioli, Peter W. McCarthy

**Affiliations:** 1 The Higher Educational Key Laboratory for Measuring and Control Technology and Instrumentations of Heilongjiang Province, Harbin University of Science and Technology, Harbin 150080, China; 2 Murdoch University Chiropractic Clinic, Murdoch University, Murdoch 6150, Australia; 3 Faculty of Life Science and Education, University of South Wales, Treforest, Pontypridd CF37 1DL, UK; 4 Faculty of Health Sciences, Durban University of Technology, Durban 1334, South Africa

**Keywords:** body area network, healthcare, human skin, low-cost sensor, physiological parameter

## Abstract

Continuous monitoring of health status has the potential to enhance the quality of life and life expectancy of people suffering from chronic illness and of the elderly. However, such systems can only come into widespread use if the cost of manufacturing is low. Advancements in material science and engineering technology have led to a significant decrease in the expense of developing healthcare monitoring devices. This review aims to investigate the progress of the use of low-cost sensors in healthcare monitoring and discusses the challenges faced when accomplishing continuous and real-time monitoring tasks. The major findings include (1) only a small number of publications (*N* = 50) have addressed the issue of healthcare monitoring applications using low-cost sensors over the past two decades; (2) the top three algorithms used to process sensor data include SA (Statistical Analysis, 30%), SVM (Support Vector Machine, 18%), and KNN (K-Nearest Neighbour, 12%); and (3) wireless communication techniques (Zigbee, Bluetooth, Wi-Fi, and RF) serve as the major data transmission tools (77%) followed by cable connection (13%) and SD card data storage (10%). Due to the small fraction (*N* = 50) of low-cost sensor-based studies among thousands of published articles about healthcare monitoring, this review not only summarises the progress of related research but calls for researchers to devote more effort to the consideration of cost reduction as well as the size of these components.

## 1. Introduction

An ageing population and an increasing number of patients with chronic diseases (e.g., diabetes, neurological disorders, and hypertension) put enormous financial stress on any healthcare system: For instance, the overall expense of treating cardiovascular diseases in the EU is around €210 billion annually [1]. In addition, hospitalisation imposes financial as well as emotional burdens on families, with frequent hospital visits being impractical for some family members or patients living in remote areas. In contrast to this is the wish of most people to remain independent of care for longer. However, this can create an added concern, namely that if a frail or an early-stage dementia patient were to fall and be left unnoticed for more than two hours, the situation could become life-threatening [2]. To address the increasing healthcare costs and increasing numbers of aged people, the WHO has recommended continuous healthcare monitoring as a cost-effective tool for use in many areas of the healthcare process from preliminary examination, preliminary diagnosis, and preliminary treatment [3], extending to remote monitoring and enabling people to safely live independently for longer. 

Health status monitoring appears critically important for any healthy population. We would like to illustrate this using a now commonplace activity: being seated. Sitting accounts for one-third of daily activity; however, unhealthy sitting postures and prolonged periods of sitting can trigger musculoskeletal disorders, such as neck pain [4]. However, if the person has coexistent health issues, sitting for prolonged periods can exacerbate these. Furthermore, in those with neurological damage that forces them to sit for prolonged periods, the accumulated pressure loading along with the increasing temperature and humidity at the body–seat interface can lead to the deterioration of epithelial and subcutaneous tissues and ultimately pressure ulcers [5]. In the case of males, it has also been reported that the rising temperature in the body–seat microenvironment has a negative impact on sperm chromatin structure [6]. The many consequences of what is now a commonplace activity have come to the awareness of seat designers, sparking interest in the research of sitting with the aim of remotely monitoring sitting patterns in real-time [7,8,9]. This would have obvious potential benefits for healthcare, such as wheelchair users with an impaired sensation of pain and/or impaired ability to adjust their own posture. Although this example illustrates a limited aspect of sensor use, it is possible to realise from it the breadth and depth of both the information and role (from solely commercial use to inclusion in the home/car in health and healthcare) that the inclusion of simple, low-cost sensors in otherwise ordinary daily objects can have. 

Consequently, various prototypes and commercial products have been designed and developed by research facilities and industrial companies. The market momentum is expected to reach nearly $140 billion by 2026 [10]. The most significant merit of healthcare monitoring is to provide real-time feedback information about health status, either to the individuals or to healthcare workers or professional personnel at a medical centre to take action before the happening of possible imminent health threats. Healthcare monitoring devices constitute a new means of tackling the issues of predicting and preventing the occurrence of chronic diseases (e.g., diabetes mellitus and heart attacks) and facilitating the patient’s own monitoring: i.e., taking ownership of their disease (diabetes mellitus, heart conditions, sleep conditions). Other areas being explored include looking after elderly people at home (e.g., monitoring life activity change in early dementia) and assisting patients to perform training and rehabilitation tasks (e.g., fitness recordings and sitting posture recognition). Of course, they also have a major role in helping people lead healthier lifestyles if they wish (e.g., heart monitoring and sleep monitoring).

As sensor technology advances, we have seen decreased expense, reduced size, and improved integration. Such advances have allowed the development of devices to monitor the health of the elderly at home with the help of the internet which significantly reduces the number of clinical visits yet retains delivery of high-quality healthcare [11]. Such devices allow the patient to monitor their own disease and other health-related data at home, facilitating patient-centred care. As the data are transmitted to the healthcare team, they can remotely monitor and offer more personalised advice. Ethically, this system has fewer issues with privacy than camera-based systems, as it is anonymised and without any unnecessary visual data that can breach privacy.

Although a great deal of literature summarising the progress of healthcare monitoring has been published in recent years, to the authors’ knowledge, none discusses the achievement based on the usage of low-cost sensors nor compares sensor cost as part of their discussion. In this review, we used two features to define the low cost: (1) clear declaration of using low-cost sensors and (2) the whole cost of the sensing component is less than 20 USD (we do not consider the price of the data processing unit, such as the high-performance remote server, in this calculation, as the data processing unit can be shared by multiple applications. Consequently, the cost for each individual node would be very low). If any of the above criteria are met, the application is categorised as using low-cost sensors.

Applying low-cost sensors to monitor human health is of particular importance to maintaining personal well-being and increasing the independence of people who lose control or sensation of body organs caused by disease/injury damage [11,12,13]. As a result, reliable low-cost healthcare monitoring can effectively reduce the economic burdens of families and release the workload of caregivers. In addition, low-cost healthcare monitoring can improve the living standards of daily life by assisting patients to live independently. 

An essential part of a typical healthcare monitoring system encompasses various types of highly integrated sensors that are capable of measuring a plethora of health metrics [13,14], including electrocardiogram [15], blood pressure [1,3], temperature and humidity [4,5,8,9], oxygen saturation, glucose level, pulse rate [14,16], and body movements [13,17]. A continuous monitoring system working in the daily environment is capable of providing real-time and non-invasive biometric signal measurement and further sets off potential disease warnings in advance, offering effective illness prevention and treatment advice or evaluating health conditions, thus reducing healthcare spending and frequent hospital visits [16].

To our best knowledge, this is the first review of studies that use low-cost sensors for healthcare monitoring over the past two decades. We have classified the various algorithms used to process sensor data into three groups from the perspective of data processing hierarchy. In addition, the performance and practical issues of wireless communication techniques have been compared to cable connections. Finally, we have summarised the future direction of healthcare monitoring using low-cost sensors with the help of word clouds.

This paper is organised as follows: Section 1 demonstrates the critical role that healthcare monitoring has played and is playing in public healthcare, emphasising the significance of using low-cost sensors when considering the monitoring of health status. Section 2 illustrates the structure of the literature search, including the inclusion/exclusion criteria used. Section 3 compares the different algorithms and data communication techniques utilised by healthcare monitoring systems. Section 4 identifies the challenges and future research directions. Finally, Section 5 concludes our findings. 

## 2. Materials and Methods

A systematic search of the literature was conducted on four internet databases: medical (PubMed); technical (IEEE Xplore and EI Village); and all science (Web of Science, WOS). Each database was searched in English only, dating from January 2002 to December 2021. To avoid studies being not included by the online retrieval search engines, we manually reviewed the references of all selected articles. The inclusion and exclusion criteria for the review were designed prior to the literature search. To be included in the literature review, the articles needed to:

1. Be published in peer-reviewed journals from January 2002 to December 2021;
2. Be written in the English language;
3. Be measured by non-invasive, low-cost sensors;
4. Be either cohort studies, cross-sectional studies, case series, or case reports with experiments on human participants (not manikins);

Three groups of terminologies were used in the literature search, and one from each group was selected to search within titles, keywords, and abstracts as follows. Group I: “healthcare”, “skin damage”, “human movement”, or “disabled”, Group II: “measur*” or “detect*” “body area network” or “monitor/monitoring”, in which the wild character “*” represents words with same alphabet. For example, “measur*” includes measure, measuring and measurement, while “detect*” involves detector, detection, and detecting as well as detect, and Group III: “optical”, “acoustic”, “electronic”, “magnetic”, “sensor”, “transducer”, “device”, “hardware”, “equipment”, or “system”.

As the electronic database system could only provide rough search results, we scrutinised all papers found using titles, keywords, and abstract information to exclude those that did not meet the inclusion criteria. Articles were excluded, for instance, if the studies were performed using expensive, commercially available medical devices (e.g., X-ray, CT, and MRI), sophisticated motion capturing systems (e.g., Vicon (Vicon Motion Systems Ltd., Oxford, UK) or Optotrak (Northern Digital Inc., Waterloo, ON, Canada), Kinect (Microsoft Corporation, Washington, DC, USA), or thermal cameras), pressure imaging systems (e.g., Xsensor Co. Calgary, AB, Canada), or implanted sensors. However, studies using cheap webcams or custom-made pressure sensors were included. If the titles and keywords did not provide sufficient information to enable a clear exclusion or inclusion decision, the abstracts or main texts would be examined as required. In addition, repeated search results (same papers listed by a different internet database) were also removed using excel-based tool kit.

Regarding the “low cost sensor” requirement, we first used the PDF reader’s embedded search function to automatically go through the whole paper; if the words “low cost sensor” or “low-cost sensor” were found, the related article would be labelled and put into the corresponding folder for further inspection. When there were no such specific words (“low cost”), we checked the price of sensors utilised by the researchers. If the whole cost of the sensing component was found to be less than 20 USD (using Taobao.com to check the price, a Chinese online purchase platform similar to eBay), this article was also labelled as meeting the criteria.

**Figure 1:**
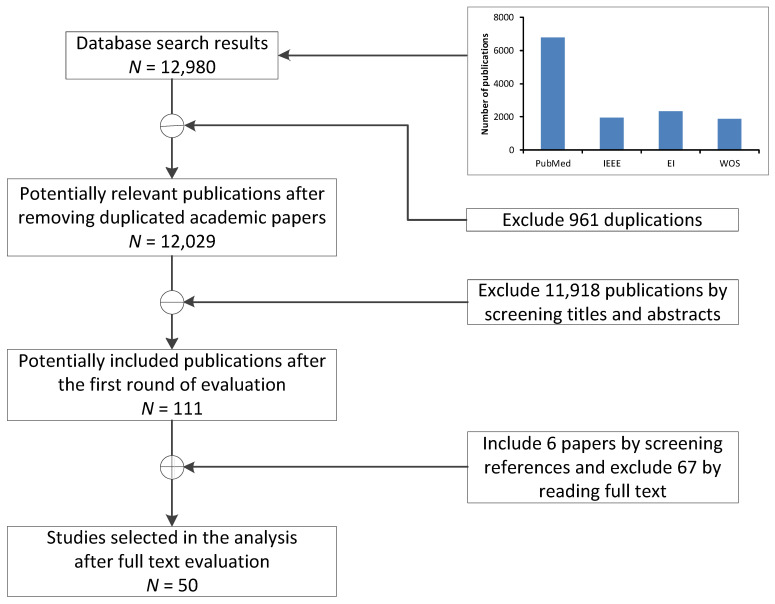
Flow chart of literature selection through the review phases. The contributions of IEEE, EI, and WOS were similar: 1963, 2356, and 1863, respectively, while PubMed contributed more than half of all publications (6798) in the initial search.

A peer-review process was applied during the final selection stages in which five reviewers participated. An agreement was reached by consistent consent among the reviewers in the few cases of disagreement. Relevant references cited by the selected papers were checked meticulously by screening titles and abstracts (Figure 1).

## 3. Results

The combination of low-cost hardware and advanced algorithms has made once hospital-only healthcare monitoring devices pervasively available at home and provided accessible living assistance to patients [18]. 

### 3.1. Health Information Measurement Using Low-Cost Sensors

In accordance with the results of the literature search (Table 1), various physiological signals (e.g., heart rate, blood oxygen, and body temperature) can be acquired using low-cost sensors made of different materials, such as capacitive, resistance, fibre optic, and electromagnetic substances. 

In some applications, sensors are closely placed on the skin (e.g., PPG and GSR) [22,23]; however, prolonged and continuous contact may trigger skin irritation or even skin diseases in the worst situation [57,58]. In addition, airborne infectious diseases can be transmitted through face-to-face contact (e.g., talking, coughing, or sneezing). A noncontact detection system with protection (e.g., wearable temperature measurement integrated into face masks) may effectively limit the spread of respiratory viruses, such as COVID-19 or seasonal influenza [20].

Among the applications of low-cost sensors used for healthcare monitoring (*n* = 50), IMU sensors account for 40% thanks to the advancement of MEMS followed by pressure detection using piezoelectric, textile capacitive, or fibre optic sensors (33%) and then vital sign measurements, such as body temperature, pulse rate, ECG, and respiration (21%).

### 3.2. Algorithms Used to Process Sensor Data

Although the physiological signals collected by sensors are different in relation to the various applications, the data processing component tends to have similarities. Generally, the first stage is referred to as data cleaning in which the raw data from the sensors undergo preliminary processing, including noise suppression, missing data interpolation, and outlier removal [12]. Environmental disturbance (e.g., body movement or breathing) usually contaminates the data and can mask or make it difficult to resolve important information (e.g., changing the original pattern/trend). In terms of noise suppression, time-domain and frequency-domain filters are two typically used methodologies, including the usage of a median filter, moving average filter, and bandpass/bandstop filter. By way of illustration, the Butterworth filter has been used to remove low frequency noise (cut-off frequency is 0.25 Hz) when using IMU sensors to acquire wrist movement activities of people suffering from spinal cord injury [33].

The second stage of sensor data processing is called feature selection. In this procedure, various methodologies are employed to derive unique characteristics from the preprocessed data. Statistical features (e.g., mean, median, skewness, and correlation [35,38,69]) can be easily derived (e.g., arithmetic mean, variance, and interquartile range minimum and maximum were used to represent upper arm and forearm movements in the therapeutic treatment [30]), while more reliable features can be extracted using more advanced time-frequency analysis techniques, such as wavelet decomposition and Fourier transforms. Compared to classic FFT, wavelet decomposition has higher computation complexity. As examples of such usage, FFT was applied to extract maximum frequency components from PPG data [22] and WT was used in the process of depicting the gait characteristics of PD patients [34]. However, features extracted using the wavelet decomposition contain both temporal and frequency information, which is more robust and accurate [34]. The selected features serve as an optimal representation of the original data by removing redundant information [70].

Once the feature selection process is complete, the final stage is to correctly classify different information into corresponding categories or predict the trend of physiological parameters. Machine learning techniques, including supervised and unsupervised learning approaches, are widely used for classification or prediction. Among the supervised algorithms, the threshold classifier is the simplest and the most efficient to discriminate binary states, such as falling detection. In comparison, SVM and DT algorithms are more complex but outperform the threshold classifier in terms of distinguishing multiple and sophisticated states. However, poor interpretation can be the weak point of advanced algorithms (e.g., SVM and DT) [70]. Regarding the unsupervised learning algorithms, input data are automatically grouped into different clusters by exploring the similarities or hidden patterns in characteristic datasets. Typical unsupervised algorithms include KNN, HMM, and hierarchical means.

**Figure 2:**
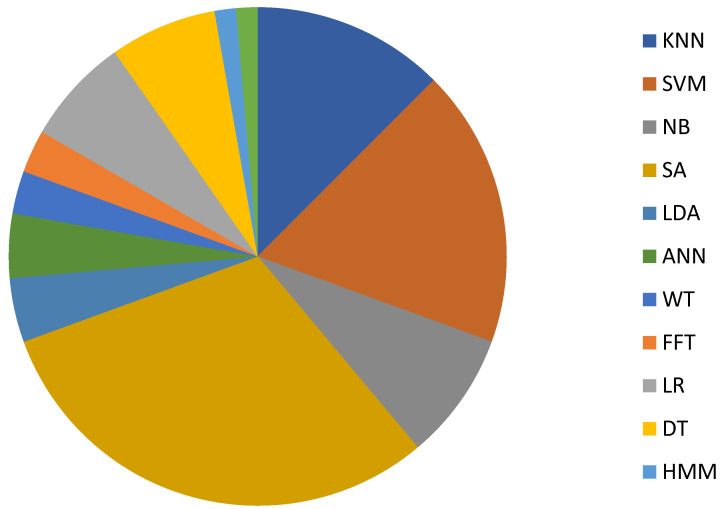
Summary of typical algorithms used for processing data acquired by low-cost sensors including LR [20], DT [21], FFT [22], SVM [22], NB [23], SA [29], WT [34], LDA [40], ANN [42], HMM [56] and KNN [65].

Among the algorithms used in healthcare monitoring (Figure 2), SA accounts for nearly one-third (31%) followed by SVM (18%) and KNN (13%). It is interesting to note that multiple methods were employed and compared corresponding to some specific applications, such as sitting posture recognitions [64,65,66,67,68]. With the advancement of computer technology, deep learning, which originated from the ANN, has emerged to become another better solution to address classification/prediction problems. A CNN is applicable to high-dimensional data while LSTM is used for one dimension in the time domain. Compared with machine learning algorithms, deep learning techniques require a much larger amount of data for training the network. In addition, labelling data in the training stage can be very tedious and time-costing, not to mention the training period being consumed by the multilayer network [9,71,72]. 

### 3.3. Techniques Used for Data Transmission and Storage

To achieve the data processing tasks mentioned in Section 3.2, sensor signals must be transmitted to high-performance computers either by cables or wirelessly. In some applications, SD memory cards are also utilised for data storage, and later offline processing is carried out after the completion of data acquisition.

Due to the inconvenience triggered by cable connections, wireless communications have become the popular option for healthcare monitoring applications (Zigbee, Bluetooth, Wi-Fi, and RF together account for 77% in comparison to 13% of cable connections and 10% of SD card usage).

**Figure 3:**
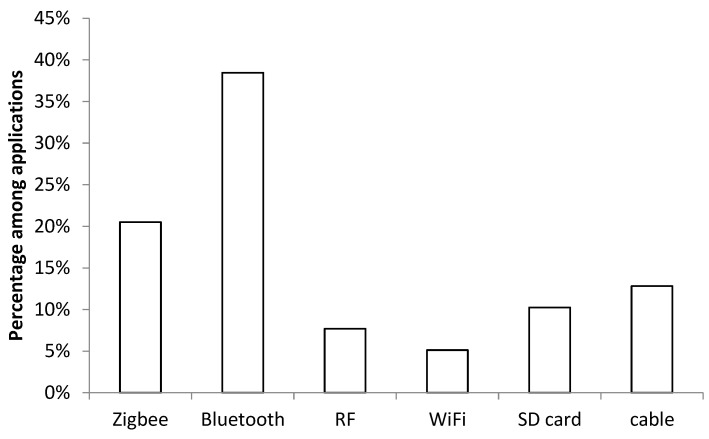
Comparison of different techniques used for sensor data transmission and storage.

Due to the merits of low power consumption and low cost, Bluetooth (based on IEEE 802.15.1 standards) and Zigbee (based on IEEE 802.15.4 standards) were the top two short-range wireless communication solutions found followed by RF and Wi-Fi-based applications (Figure 3).

Zigbee provides the possibility of connecting up to 65,536 nodes with the help of a mesh networking topology [15]. Ad hoc and star connections are alternative topological structures used by Zigbee, while Bluetooth only supports point-to-point and point-to-multipoint expansions. In comparison, Wi-Fi has more topological structures, such as ad hoc, bridge, and repeater [73].

The working range of Zigbee is from 10 to 100 m, which is the same as Bluetooth communication. Although the maximum working range of Wi-Fi is within 10 m, the data transfer rate is up to 6.75 Gbps, which is much faster than Bluetooth (≤2.1 Mbps) and Zigbee (≤250 Kbps).

RF tags have been developed to replace wrist-banded bar codes for locating patients and storing medical information [67,73]. To fully explore the capabilities of wireless communication techniques, different modules are combined to implement a specific task in some situations. For example, Zigbee and Wi-Fi are utilised to transmit physiological signals to physicians [15] and monitor the ambulatory activities of older people in fall prevention [67].

### 3.4. Research Trend of Low-Cost Sensor-Based Healthcare Monitoring

**Figure 4:**
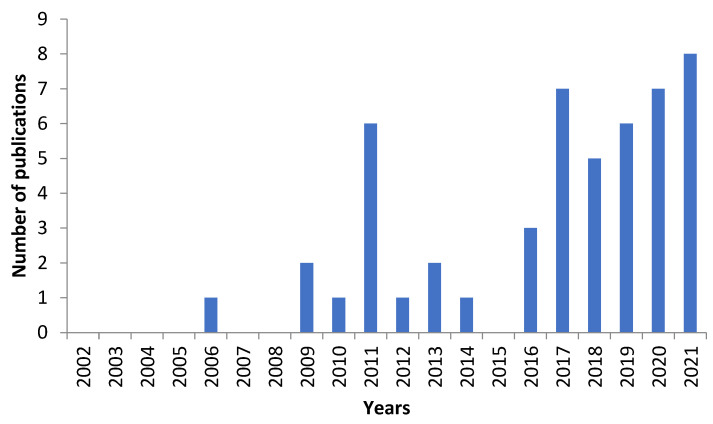
Number of publications between 2002 and 2021 using low-cost sensors for healthcare monitoring.

The number of publications related to healthcare monitoring has increased significantly over the past two decades (Figure 4). Particularly, there has been a rapid increase in the past ten years (between 2012 and 2021) with publications quadrupling (40/50) in comparison with 2002–2011 (10/50). Although similar research activities were conducted between 2002 and 2011, most of the experiments employed commercially available products with a price > 4000 USD [38,39].

**Figure 5:**
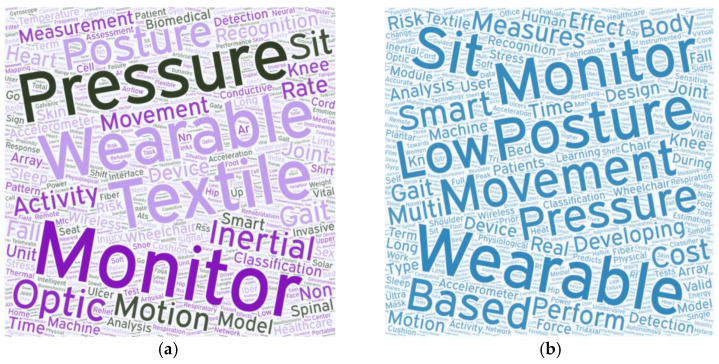
Word cloud of keywords and titles: (**a**) keywords and (**b**) title. The larger the font is, the more frequently the word appears. (**a**) Word clouds of the keywords from the selected publications and (**b**) word clouds of the article titles from the selected publications.

The frequency of vocabulary appearing in keywords or titles was counted except for several online publications that did not provide keywords [24,26,27,34,61,62]. Among the top ten most frequently appearing words, wearable, monitor, posture, movement, and pressure appear in both article titles and keywords (Figure 5).

## 4. Discussion

Compared with previous publications, this review focuses on the applications of using low-cost sensors to monitor health status. Below, we discuss the issues impacting sensor usage in healthcare monitoring, as this will help readers appreciate the synthesis presented above.

### 4.1. Challenges Faced by Low-Cost Sensor-Based Healthcare Monitoring

(1) **Concerns about a data privacy breach**. With the introduction of cloud computing and data sharing among Internet servers, third-party service providers may unintentionally disclose personal health information (e.g., attacks by malicious ransomware and password hacking). After that, “digital thieves” could deduce an individual’s daily activities and lifestyles and consequently put the privacy and security of the users and their property at stake; more usually, this type of data access could be used for advertising or marketing purposes [74,75,76]. Thus, a solution to the issue of information privacy and security appears crucial.

However, embedding sophisticated security schemes into a low-cost healthcare device is also full of challenges. Furthermore, studies show that a large amount of healthcare devices do not follow well-established design guidelines or meet the legal regulations imposed by administration agencies, such as the General Data Protection Regulation of the EU and the Health Insurance Portability and Accountability Act of the US, thus endangering the privacy of millions of customers [76,77,78,79]. A review of 24,405 health-related apps, for instance, concluded 95.63% of the iOS- and Android-based applications were at risk of information leakage and privacy infringement, whereas 11.67% of them would be susceptible to the highest potential damage [79].

(2) **Lack of standardised testing protocols**. In the process of evaluating low-cost sensor-based healthcare monitoring devices, a predominant difference is the shortage of adequate clinical trials comparing low-cost sensor systems to currently used, commercially available products. Although participants were recruited in some studies, the numbers were not sufficient to reach a confident conclusion [7,80,81].

Longer test periods are also needed to investigate the potential side effects on users. For example, some healthcare monitoring devices are to be worn by users for such a long time that some form of comfort index should be included in the evaluation process, especially for products targeting patients and those potential users in the senior age group. However, the comfort investigation heavily relies on the user’s perception and can easily be influenced by other factors, such as ambient environment, mood, and aesthetics [57,58].

(3) **Shortage of sustainable energy supply**. Large amounts of wearable medical devices rely on internal power sources (e.g., batteries), which obviously will require regular recharging/replacement to ensure an appropriate constant power supply [10].

Although supercapacitors enable a large amount of energy storage, self-discharge in an idle state or leakage due to environmental changes (e.g., wetness or high temperature) can cause the loss of stored energy. In addition, efficiently managing power consumption (e.g., screen off in an idle state) is another aspect to be helpful in extending the period of battery usage. If battery management software is to be incorporated, this may impact power consumption.

### 4.2. Future Work

Although reducing the cost of healthcare monitoring is a major concern, there is a range of additional challenges which have been confronted, thus helping to illuminate the issues future research needs to overcome.

(1) Individual’s health information is deemed so sensitive by nature that sufficient protection is of substantial importance both legally and technically [76]. Thus, advanced encryption/decryption algorithms and authentication mechanisms would be required to ensure sensitive data (e.g., patient symptoms, activity profile) is transferred and communicated safely and reliably [14].

Apart from random number-based cryptographic algorithms (e.g., symmetrical and asymmetrical cryptography), biometric authentication has become an emerging research topic in the field of security and privacy protection, which include two types of biometrics: behavioural biometric traits, including signature, voice, gait, ECG, and keystrokes, and physical biometric traits, including fingerprint, palm print, face, retina/iris, hand geometry, ear shape, body odour, skin surface venous patterns, and DNA [18]. In order to overcome the lower accuracy of single biometric authentication, feature fusion techniques have been adopted by integrating multiple biometric characteristics to improve accuracy in different scenarios [18]. Some researchers captured gait biometrics (e.g., walking patterns) to generate random numbers with the help of smart phone-based inertial sensors [82,83]. Despite the uniqueness and distinctiveness of biometric features, the performance may degrade due to change/damage resulting from senescence in the individual or physical injuries [18,84,85]. In addition, the cost of hardware and software still impose a heavy financial burden on acquiring biometric traits: for instance, the face and retina require high-quality cameras/scanners and when using finger or palm prints as authenticating characteristics accuracy is low for wet hands [18]. As a further consideration, future systems would benefit from having some self-repairing capability (e.g., post viral attack) to facilitate the resumption of operation with minimal need for human intervention [18,86,87].

Another research aspect could be to develop an agreed framework for assessing the security strength of healthcare monitoring systems [88,89]. The framework should consider both the diverse facets of the security and privacy policies as well as easy access by authorised entities (e.g., clinic doctors and devices) and users. It would be inconvenient, for instance, to ask permission from the user every time when a data access request is made; although, there could be an ethical prerequisite to do so. Therefore, algorithms should be intelligent enough to automatically decide which data can be shared without permissions and which third parties can be granted access rights [18]. From the technical perspective, fully developed security systems should be evaluated using standardised protocols, such as the seven attack surfaces and eight security analysis criteria [76].

(2) Regarding the standards to evaluate the healthcare systems, the Declaration of Helsinki provides a road map on experiments involving humans, with publication requiring freely given informed consent in an experiment with approval from an appropriate internal review board or ethics committee [90,91]. In addition to the ethical probity of the experimental protocol, statistical validity needs to be considered, with the known minimum participation number and recognition of the target population being important factors that should be considered when recruiting volunteers. Recognition of the appropriate comparison methodology for clinical studies is important, so the production of more randomised clinical trials would benefit sensor developments, attracting the attention of clinicians to the work. Among the searched literature, only four articles investigated daily activities using low-cost sensors worn by both healthy participants and PD patients [32,33,34,48]. Additionally, another publication studied the biomechanical characteristics of marathon athletes using wearable IMU sensors [36].

As medical devices are intended for use without affecting the patient’s daily activities, discomfort may not only affect the user but may also cause unintended harm to users (e.g., pressure ulcers caused by uncomfortable seat cushions or bed mattresses). To effectively study the perception of comfort, some standardised protocols [7,80] should be developed to minimise the impact of the surrounding environment on the subjects. In studies of sitting comfort, for example, researchers may replace the subjective questionnaire-based feedback with objective parameters, such as interface pressure, temperature, and relative humidity, and indicators of discomfort, such as fidgeting, which may help reduce the required number of participants and be less time-consuming, more objective, and obviously cost-saving [69].

Since some health conditions require continuous monitoring, sensor output constancy should be considered, especially for ICU patients [92,93,94]. For instance, drift in calibration to prolonged use, such as the accumulation of individually minuscule errors in accelerometers, may lead to less reliable output data. In addition, improper operations of healthcare monitoring devices can cause unexpected hazards [95]. The current manufacturers’ data sheets do not necessarily give stable data in the environment of intended use. It is therefore important for researchers to consider publishing data from repetitive recalibration using a calibrated chamber certificated as traceable to an international standard. To overcome these difficulties, self-checking and automatic recalibration algorithms should be investigated to compensate for any drift phenomena [12].

(3) To overcome the need for frequent recharging or regular replacement of batteries, energy scavenging or harvesting is a promising research direction to extend lifespan or enhance battery performance. Energy harvesting is the technique of transforming ambient (e.g., light source or electromagnetic field) or biochemical (e.g., body thermal or mechanical energy) energies into applicable electrical power. Among them, photovoltaic cells, which absorb solar or indoor illumination to generate electricity, are becoming widely utilised in products.

In addition to advances in power density, extensibility, and sensitivity [10,96,97], flexible materials have been used to produce solar-powered cells, which can be integrated into clothing ideal for wearable applications [98]. Biomechanical (e.g., muscular movement) and biochemical (e.g., glucose) energies from human bodies offer other solutions for independently powering healthcare devices. For instance, walking is an efficient means of collecting human biomechanical energy: By inserting a piezoelectric ceramic transducer in the shoes, studies have shown that nearly 70 W of electric power can be generated from an adult walking at normal speed [99]. Scavenging electromagnetic waves, including Wi-Fi, radio, and television broadcasting, may also be considered as another potential option for electricity generation.

However, energy harvesting faces some technical bottlenecks, including (1) maintaining optimal orientation, incidence angle, and location of photovoltaic cells for solar energy [100], (2) the low energy conversion rate for piezoelectric/turboelectric generators [101,102], and (3) the relative inflexibility and size of effective electromagnetic harvesters [10,100]. To overcome these difficulties, energy prediction has been growing in importance, targeting more efficient usage of the limited battery power available. The current energy prediction methodologies can be divided into several categories: statistical (e.g., exponentially weighted moving average model) [103,104], stochastic (e.g., Markov model) [10,105], and machine learning algorithms (e.g., supervised and unsupervised learning) as well as the deep learning approaches [10].

### 4.3. Limitations

As the preparation of this paper started in the middle of 2022, our literature survey targeted the achievements of the past two decades (January 2002 to December 2021). However, we considered it important to include a synopsis of the academic articles published in 2022. To avoid duplications between different search engines and improve efficiency, we concentrated on the progress in the engineering field by using the EI database as the source of the literature search. The findings show that the integration of multiple MEMS sensors and IoT-based advanced algorithms still serves as the major tool to monitor health status [106,107,108]. Regarding noncontact measurement, Wi-Fi has been increasingly used as part of systems designed to monitor daily activities and detect accidental falls, with an average accuracy of 96.9% and 93.3%, respectively [109]. Although our search on the 2022 publication is not presented as a full literature review, the results depict the trends and challenges of using low-cost sensor-based healthcare monitoring. Essentially, similar conclusions can be drawn between 2022 publications and those from the past two decades (January 2002 to December 2021).

Although we did not include the Scopus database in our search, we searched publication reference lists in an attempt to capture those articles not immediately picked up by our search strings. It still remains possible that we missed some relevant articles (as with any review) and that they were included in Scopus; therefore, we consider this a potential limitation with apologies to those we did not include. This aside, we did find a large number of duplications across the databases (please see Figure 1), so results from the current four databases (PubMed, IEEE, EI and WOS) illustrate the progress of development and incorporation of low-cost sensors in healthcare research and the intended direction of future incorporation into healthcare products.

## 5. Conclusions

This review investigates both the development and application of low-cost sensors in health status monitoring to facilitate independent living and thus increase the quality of life in the elderly and those with a chronic debilitating disease.

Among the included research (*n* = 50), 40% of articles studied systems designed to recognise particular abnormal activities that are helpful in preventing (or recognising) accidental falls and causes, such as muscle fatigue. Another interesting application is to replace questionnaires (subjective sensation) with low-cost sensors creating an objective evaluation for sitting/sleeping comfort evaluation and removing the subjects/patients’ subconscious need to please the researcher (who they perceive as wanting to help them). The objective measurement not only avoids subjective bias but also provides physiological parameters useful in preventing skin illness (e.g., pressure ulcers) in those without sensory feedback.

## Figures and Tables

**Table 1:** Overview of studies on health information measurement using different low-cost sensors.

Reference	Participant	Sensors	Measurement	Evaluation
[19]	A male subject	Accelerometer, cardiac activity electrodes, and inductive plethysmographic sensor mounted on the T-shirt	Respiration rate, heart rate, and movement of the body	Absolute mean percent differences are heart rates < 2% and respiration rates < 5%.
[20]	*N* = 14	Temperature sensor embedded in KN95 mask	Respiration rate detection is based on the measurement of temperature variations through the vent holes of the mask and results can be applied to COVID-19 prevention	MAE = 0.449 BPM
[21]	*N* = 10 (M = 7, F = 3) Age = 23.8 ± 0.84 years Height = 173.1 ± 6.9 cm Body mass = 66.2 ± 6.9 kg	IMU and pressure sensor attached to insole and on legs	Motion gait recognition	Accuracy = 99.96%
[22]	*N* = 16 (M = 10, F = 6) Age = 20–54 years	Temperature sensor, pulse oximetry sensor, accelerometer, and GSR sensor attached to the upper arm	Perspiration measurement, activity recognition, skin temperature, blood oxygen saturation, heart rate	Accuracy = 87.5% (activity recognition)
[23]	*N* = 16	PPG and GSR sensors attached to fingertip and wrist	Stress index	Accuracy = 85.3%
[24]	*N* = 10 (M = 7, F = 3) Age = 26 ± 3 years Height = 165 ± 8 cm Body mass = 60 ± 10 kg	Accelerometer and angular velocity sensors attached to low back and leg	Postural detection	Statistical results show IMU sensors are suitable for detection and evaluation of anticipatory postural adjustments
[25]	*N* = 12 (M = 7, F = 5) Age = 24.91 ± 2.74 years Height = 166.91 ± 6.76 cm BMI = 61.41 ± 8.69	Textile capacitive proximity sensor placed under the feet	Gait measurement	Error rate of stride <1% Correlation coefficient between the reference sensor and the textile sensor is 0.865
[26]	*N* = 5	ECG sensor, pulse oximeter, temperature sensor attached to fingertip and body	Heart rate, respiratory rate, blood oxygen saturation, and body temperature	Accuracy = 99.26%
[27]	*N* = 25 (M = 10, F = 15) Averaged age = 56.25 years	IMU sensors attached to wrist or elbow	Shoulder joint mobility	Correlation coefficients between IMU and the traditional method are 0.997, 0.978, 0.897, and 0.984 for flexion, abduction, external rotation, and internal rotation, respectively
[28]	*N* = 12 Age = 18–57 years	Six 3-axis accelerometers and 12 gyroscopes placed on neck, left wrist, right wrist, waist, left leg, and right leg	Posture recognition (sitting, standing, walking, and lying)	Accuracy = 99.72%
[29]	*N* = 100 (M = 50, F = 50) Age = 33 ± 6 years (M) Age = 32 ± 9 years (F) BMI = 23.1 ± 2.7 (M) BMI = 20.5 ± 2.1 (F)	10 plantar pressure sensors underneath the insole	Foot pressure values during walking and standing	Statistical results show no significant difference between men and women in centre of pressure, while women exhibit higher peak pressure on the hallux, toes, forefoot, and medial aspect of the foot
[30]	*N* = 5	Wearable inertial sensors attached to upper arm and forearm	Therapeutic movement measurement guided by therapists aiming to recover after the motion impairment	Specificity = 100% Sensitivity = 100%
[31]	*N* = 3 (M = 3, F = 0) Age = 24.7 ± 2.4 years Height = 174.3 ±4.2 cm Body mass = 65.3 ± 7.0 kg	A triaxis accelerometer and three single-axis gyro sensors attached to left/right thigh and left/right shank	Angular velocity and acceleration	Hip joint angle (flexion–extension) RMSE = 8.72, ADE = 6.57, CC = 0.88, PVU = 20.05% Hip joint angle (abduction–adduction) RMSE = 4.96, ADE = 3.30, CC = 0.72, PVU = 39.29% Knee joint angle (flexion–extension) RMSE = 6.79, ADE = 4.65, CC = 0.92, PVU = 14.60%
[32]	*N* = 116 Age = 69 ± 18 years BMI = 27 ± 6	A piezoelectric sensor under the mattress	Respiratory rate, heart rate, and motion level	Specificity = 93%. Sensitivity = 85%
[33]	*N* = 30 (M = 21, F = 10, where 11 paraplegic and 19 tetraplegic subjects) Age = 46.43 ± 16.91 years	3D accelerometer and 3D gyroscope attached on each wrist and the right wheel of the wheelchair	Acceleration and peak velocity	Accuracy = 90%
[34]	PD patients *N* = 48 (M = 25, F = 23) Age = 70.61 ± 9.51 years Healthy people *N* = 40 (M = 22, F = 18); Age = 69.36 ± 7.42 years	Accelerometer attached to left/right knee	Gait characteristics of PD patients and healthy people	Specificity = 90.91%. Sensitivity = 92.86% Accuracy = 88.46%
[35]	*N* = 28 (M = 22, F = 6) Age = 41 ± 12 years Height = 70 ± 4 inch Body mass = 175 ± 43 lb	Eight force sensors placed on wheelchair cushions	Peak pressure index, weight shift frequency, pressure relief frequency, in-seat activity frequency	Statistical results show force sensors can effectively monitor wheelchair users’ movements
[36]	*N* = 27 (M = 12, F = 15) Age = 50.24 ± 12.99 years (M) Age = 40.93 ± 10.27 years (F) Height = 174.88 ± 10.25 cm (M) Height = 160.53 ± 4.31 cm (F) Body mass = 79.03 ± 11.99 kg (M) Body mass = 58.23 ± 7.83 kg (F)	IMU sensor clipped to the back of marathon runners’ shorts	Step frequency, change in forward velocity, vertical oscillation, side-to-side movement of the pelvis, side-to-side drop of the pelvis, ground contact time	Statistical analysis shows IMU-based biomechanical indices can be used to detect fatigue in marathon runners
[37]	*N* = 7	Eight hetero-core fibre optic pressure sensors placed on bed cushion	Respiration rate	Sensitivity = 0.05–0.2 dB
[38]	*N* = 7	Pulse oximeter and heart rate sensor, thermometer, and ECG sensor	Oxygen saturation (SpO2), heart rate, body temperature, ECG	Accuracy = 1.02% (blood oxygen saturation detection) Accuracy = 0.51% (body temperature measurement)
[39]	*N* = 8	IMU attached to the arm	Measure shoulder and elbow joint angles to continuously monitor human movement	Average correlation coefficient is >0.95 between the inertial tracker and the optical reference system. RMSE < 8 º (averaged value of eight subjects for all tasks) Peak-to-peak error < 12 º
[40]	*N* = 72 (M = 39, F = 33) BMI = 28.74 ± 4.99 (HFR) BMI = 28.7 ± 4.81 (LFR) Age = 71.87 ± 6.45 years (HFR) Age = 63.47 ± 8.74 years (LFR)	Four inertial sensors attached above and below each knee	Completion times for each test subactivity, joint range of motion, and flexion/extension velocities and accelerations	Accuracy = 90% Sensitivity = 94% Specificity = 59%
[41]	*N* = 5 (M = 1, F = 4) Age = 24.8 ± 3.5 years	Conductive textile sensors For lateral bending measurement, sensors are placed on both sides under the angle of the mandible and in correspondence with the trapezius scapula insertion; For axial rotation motion, sensors are placed on both sides on the anterior part of angle of the mandible and in correspondence with the trapezius muscle; For flexion–extension movement, one sensor is placed between the hyoid bone and the sternum (extension), the other between C2 and C7 vertebrae (flexion)	Measure the angle (in degrees) of lateral bending, rotation, and flexion–extension of cervical spine movement	RMSE values of lateral bending, axial rotation, and flexion/extension of neck were 6.04 ± 0.67, 10.16 ± 2.11, and 12.31 ± 3.22, respectively
[42]	*N* = 13 (M = 13, F = 0) Age = 26.1 ± 2.9 years Height = 178.7 ± 5.5 cm Body mass = 78.4 ± 5.9 kg	Two identical, custom-built, six-degrees-of-freedom IMUs (accelerometer and gyroscope) attached to the right thing and shank via a knee sleeve	Knee joint forces	Accuracy Vertical force: RMSE = 19.1% ± 4.0%, anterior–posterior: RMSE = 21.8% ± 2.6%, medial–lateral: RMSE = 38.0% ± 6.1%
[43]	Young subjects: *N* = 21 Aged = 28.3 ± 6.8 years Body mass = 67.2 ± 9.6 kg Height = 1.70 ± 0.04 m Fallers: *N* = 16 Aged = 67.2 ± 6.7 years Body mass = 64.3 ± 12.0 kg Height = 1.58 ± 0.07 m	Four load cells fixed to the chair	Force between sitting and standing swap	Error < 10%
[44]	*N* = 6 (five aged 22 to 23 and one aged 60)	Two dual-axis accelerometers orthogonally mounted on the waist	Daily activity detection	Accuracy = 90.8% (12 tasks) Accuracy = 94.1% (postural recognition) Accuracy = 83.3% (walking recognition) Accuracy = 95.6% (falling detection)
[45]	*N* = 2 (M = 1, F = 1) Female: 1.58 m in height, 53 kg in body mass, 25 years; Male: 1.77 m in height, 75 kg in body mass, 24 years	Three wireless transceiver modules fixed to the arm/leg with an elastic band	Arm and leg movements	Matching rate using two features: 70% and 80% for females and males, respectively. Matching rate using five features: 90% and 100% for females and males, respectively.
[46]	*N* = 8 Age = 20–35 years	48 fibre-optic pressure sensors placed below the mattress	Breathing rate, torso movement, sleep monitoring	Sensitivity = 71% Specificity = 87%
[47]	*N* = 9 (M = 3, F = 6) Age = 23.3 ± 2.5 years, Body mass = 55.4 ± 8.5 kg, Height = 1.60 ± 0.08 m	One triaxial accelerometer and three uniaxial gyroscopes were secured onto the back of the subjects	Angular measurements during trunk movement; trunk postural change	Correlation coefficients between the Vicon video capture system and sensors: >0.994 for dynamic tilting measurements and >0.776 for trunk postural measurements
[48]	*N* = 8 (four healthy subjects and four stroke survivors)	Triaxial accelerometers and triaxial gyroscopes worn on waist	Arm movement	Healthy people: Accuracy = 86% (accelerometer) and 72% (gyroscope) Stroke patients: Accuracy = 67% (accelerometer) and 60% (gyroscope)
[49]	*N* = 8 (M = 2, F = 6) Age = 30 ± 5 years Body mass = 70 ± 15 kg	IMU sensors placed on a glove worn by the driver	Stress indicators: emergency braking and rapid turning	Accuracy = 94.78%
[50]	*N* = 17 (M = 8, F = 9) Age = 21.9 ± 3.7 years	IMU sensors attached the right leg to Velcro strap	Knee flexion/extension angles	RMSE = 5.0º ± 1.0º MAE = 3.9º ± 0.8º
[51]	*N* = 70 Age = 18–86 years	IMU, temperature, pressure and GSR sensor attached to thigh with Velcro strap	Acceleration, angular velocity, skin temperature, muscle pressure, and sweat rate	The concordance correlation coefficient is 0.96 in comparison with the video motion analysis system. The highest estimation error for stride length was 4.81 cm (3.3%), and the mean error (*N* = 10) was 2.48 cm (1.7%). For gait speed, the estimation error < 3.8% (5.10 cm/s) and the mean error was 2.1%.
[52]	*N* = 8 (M = 5, F = 3) Age = 21–24 years	Piezoelectric sensor fixed to arms by bandage	Hand and wrist movements	Accuracy = 96.1% (LDA) Accuracy = 94.8% (ANN)
[53]	*N* = 10 Age = 21–36 years Height = 1.48–1.89 m Body mass = 46.7–91.0 kg	Five FSR sensors attach to the foot surface	Ground reaction forces	The correlations between FSR-based system and the gold standard force plate are 0.74–0.84. For hopping, the maximum GRF difference between FSR-based system and the gold standard force plate ranged from −6% to +14%.
[54]	*N* = 5 (M = 5, F = 0) Age = 31 ± 5 years Height = 170 ± 4.6 cm Body mass = 71.2 ± 4.2 kg	7 × 5 conductive textile sensors attached to leg	Knee angle during flexion–extension movements	MAE = 17.54 º RMSE = 18.82 º
[55]	*N* = 7 (M = 4, F = 3) Age = 21–60 years	64 × 128 pressure-sensitive e-textile sensors placed on bed	Respiration rate, leg movement	Precision = 70.3% Recall = 71.1%
[56]	*N* = 8 Age = 25 ± 3 years Body mass = 61 ± 19 kg	Triaxial accelerometer worn on the body	Falling detection	Sensitivity = 100% Specificity= 100%
[57]	*N* = 8 (M = 4, F = 4) Age = 23.6 ± 1.3 years Height = 1.69 ± 0.08 m Body mass = 56.2 ± 10.3 kg	Temperature sensor arrays fixed on the seat	Body–seat interface temperature measurement	Temperature field at the contact surface was not uniformly distributed. Heating rates = 1.7 ± 0.4 °C/min (fabric cover + foam) Heating rates = 1.6 ± 0.2 °C/min (wood) Heating rates = 1.7 ± 0.2 °C/min (leatherette cover + foam)
[58]	*N* = 78 (M = 39, F = 39) Participants equally divided into three groups (*n* = 26) Group 1 Age = 21.9 ± 1.8 years BMI = 21.6 ± 2.8 kg/m^2^ Group 2 Age = 22.5 ± 2.4 years BMI = 22.2 ± 3.8 kg/m^2^ Group 3 Age = 22.2 ± 3.8 years BMI = 21.7 ± 2.1 kg/m^2^	Four digital humidity and temperature sensors placed under the ischial tuberosities and thighs bilaterally	Skin temperature and relative humidity	Temperature difference after two hours: 3.9 ± 1.4 °C (ischial tuberosities) and 5.6 ± 1.3 °C (thighs), 2.8 ± 1.7 °C (ischial tuberosities) and 4.7 ± 1.4 °C (thighs), 3.9 ± 1.3 °C (ischial tuberosities) and 6.3 ± 1.1 °C (thighs) for air-filled rubber, foam–fluid hybrid and medium density foam, respectively. No significant difference in relative humidity between different cushions
[59]	*N* = 5 (F = 3, M = 2) Age = 33 ± 8 years Height = 180 ± 10 cm Body mass = 70 ± 21 kg	Flexible screen-printed piezoresistive sensors	Four sitting posture recognition	Accuracy = 80%.
[60]	*N* = 12 (M = 7, F = 5) Age = 22–36 years BMI = 16–34 kg/m^2^	FSR sensors (seven on seat pan and 5 on backrest)	Five sitting posture recognition	Accuracy = 96.85%
[61]	*N* = 41 (M = 25, F = 16) Age = 24–64 years Height = 160–200 cm Body mass = 53–126 kg	FSR sensors (10 on seat pan 4 on backrest, 2 on armrest)	Seven sitting posture recognition	Accuracy = 98%
[62]	*N* = 9 Age = 59.7 ± 24.2 years Height = 1.76 ± 0.10 m Body mass = 38.78 ± 4.94 kg	Customised piezoresistive sensors (eight sensors on seat pan and eight on backrest)	12 sitting posture recognition	Repeatability and replicability of the system are evaluated. The total cost of the system is <150 USD in comparison to commercial products with a price of ~7000 USD.
[63]	*N* = 25 (M = 15, F = 10)	Customised fibre-based yarn coated with piezoelectric polymer placed on seat	Seven sitting posture recognition	Accuracy = 85.9%
[64]	*N* = 9 (M = 6, F = 3)	Customised textile pressure sensors placed on seat	16 sitting posture recognition	Accuracy = 82%
[65]	*N* = 36 (M = 21, F = 15) Age = 26.7 ± 2.0 years (M) Age = 25.0 ± 2.3 years (F) Height = 175.9 ± 6.4 cm (M) Height = 162.8 ± 4.6 cm (F) Body mass = 77.1 ± 15.0 kg (M) Body mass = 51.4 ± 4.3 kg (F)	Six FSR sensors embedded in the seat cushion and six IRD sensors placed in the seatback	11 sitting posture classification	Accuracy = 92%
[66]	*N* = 8 (M = 8, F = 0) Age = 24–40 years	Two IMU sensors placed on the lower and upper arms (near the wrist and elbow joints), respectively	Movement of upper limbs	Angle error < 3º Position error < 9 mm
[67]	*N* = 10 Age = 19–28 years Height = 155–187 cm Body mass = 46–70 kg	Three RF sensors placed on the back of the subjects (thoracic, thoracolumbar, and lumbar regions) at the distance of 10 cm each	Sitting posture recognition	Accuracy = 98.83%
[68]	*N* = 19 (M = 14, F = 5) Age = 22–58 years	Two FSR sheets placed on seat pan (9 × 9) and backrest (10 × 9)	15 sitting postures	Accuracy = 88.52%

## Data Availability

This article has no additional data.

## Abbreviations

ADE	Absolute Deviation of Error
AI	Artificial Intelligence
ANN	Artificial Neural Networks
BMI	Body Mass Index
BPM	Breaths Per Minute
°C	Degrees Celsius
CC	Correlation Coefficient
cm	Centimetre
cm/s	Centimetre Per Second
CNN	Convolution Neural Network
dB	Decibel
DNA	Deoxyribonucleic Acid
DT	Decision Tree
ECG	Electro Cardiogram
EI	Engineering Index
F	Female
FFT	Fast Fourier Transform
FSR	Force Sensitive Resistor
GPU	Graphic Processing Unit
GRF	Ground Reaction Force
GSR	Galvanic Skin Response
H-IoT	Health-Internet of Things
HMM	Hidden Markov Model
ICU	Intensive Care Unit
HFR	High Fall Risk
IEEE	Institute of Electrical and Electronics Engineers
IMU	Inertial Measurement Unit
IR	Infrared Reflective Distance
IRD	Infrared Reflective Distance
Kg	Kilograms
KNN	K-Nearest Neighbour
LDA	Linear Discriminant Analysis
LFR	Low Fall Risk
LSTM	Long Short-Term Memory
M	Male
m	Meters
MAE	Mean Absolute Error
MEMS	Micro-Electro-Mechanical System
*N*	Number
N/A	Not Applicable
NB	Naïve Bayes
PD	Parkinson Disease
PDF	Portable Document Format
*PPG*	*Photoplethysmography*
PVU	Percentage of Variance Unexplained
RF	Radio Frequency
RMSE	Root Mean Square Error
SA	Statistical Analysis
SD	Secure Digital
SVM	Support Vector Machine
USD	United States Dollars
WHO	World Health Organisation
